# Supplementary material for: Smooth Interpolating Curves with Local Control and Monotone Alternating Curvature
Source: Comput Graph Forum. 2022 Oct 6;41(5):25–38. doi: 10.1111/cgf.14600 (PMC9827861; doi:10.1111/cgf.14600)
Supplement: Supplementary file 1 — Supplement Material [file CGF-41-25-s001.zip › Local-Smooth-Interpolating-MonoCurvature/extern/clothoids/submodules/quarticRootsFlocke/docs/api-cpp/program_listing_file_PolynomialRoots.hh.html]

Program Listing for File PolynomialRoots.hh — Quartic Roots v1.0.0

### Navigation

- index
- toc
- Quartic Roots »
- Program Listing for File PolynomialRoots.hh

# Program Listing for File PolynomialRoots.hh¶

↰ Return to documentation for file (`PolynomialRoots.hh`)

```
/*--------------------------------------------------------------------------*\
 |                                                                          |
 |  Copyright (C) 2014                                                      |
 |                                                                          |
 |         , __                 , __                                        |
 |        /|/  \               /|/  \                                       |
 |         | __/ _   ,_         | __/ _   ,_                                |
 |         |   \|/  /  |  |   | |   \|/  /  |  |   |                        |
 |         |(__/|__/   |_/ \_/|/|(__/|__/   |_/ \_/|/                       |
 |                           /|                   /|                        |
 |                           \|                   \|                        |
 |                                                                          |
 |      Enrico Bertolazzi                                                   |
 |      Dipartimento di Ingegneria Industriale                              |
 |      Universita` degli Studi di Trento                                   |
 |      email: enrico.bertolazzi@unitn.it                                   |
 |                                                                          |
\*--------------------------------------------------------------------------*/

#ifndef POLYNOMIAL_ROOTS_HH
#define POLYNOMIAL_ROOTS_HH

#include <cmath>
#include <cfloat>
#include <complex>
#include <iostream>

namespace PolynomialRoots {

  typedef double valueType;
  typedef int    indexType;
  typedef std::complex<valueType> complexType;

  #ifndef DOXYGEN_SHOULD_SKIP_THIS

  static
  inline
  bool
  isZero( valueType x )
  { return FP_ZERO == std::fpclassify(x); }

  static
  inline
  bool
  isInfinite( valueType x )
  { return FP_INFINITE == std::fpclassify(x); }

  static
  inline
  bool
  isNaN( valueType x )
  { return FP_NAN == std::fpclassify(x); }

  static
  inline
  bool
  isRegular( valueType x )
  { return !( FP_INFINITE == std::fpclassify(x) ||
              FP_NAN      == std::fpclassify(x) ); }

  valueType
  evalPoly(
    valueType const op[],
    indexType       Degree,
    valueType       x
  );

  void
  evalPolyDPoly(
    valueType const op[],
    indexType       Degree,
    valueType       x,
    valueType     & p,
    valueType     & dp
  );

  bool
  NewtonStep(
    valueType const op[],
    indexType       Degree,
    valueType     & x
  );

  std::complex<valueType>
  evalPolyC(
    valueType const                 op[],
    indexType                       Degree,
    std::complex<valueType> const & x
  );

  #endif

  int
  roots(
    valueType const * op,
    indexType         Degree,
    valueType       * zeror,
    valueType       * zeroi
  );

  // - - - - - - - - - - - - - - - - - - - - - - - - - - - - - - - - - - -
  /*\
   |    ___                  _           _   _
   |   / _ \ _   _  __ _  __| |_ __ __ _| |_(_) ___
   |  | | | | | | |/ _` |/ _` | '__/ _` | __| |/ __|
   |  | |_| | |_| | (_| | (_| | | | (_| | |_| | (__
   |   \__\_\\__,_|\__,_|\__,_|_|  \__,_|\__|_|\___|
   |
   |  A * x^2 + B * x + C
  \*/
  class Quadratic {
    valueType ABC[3];
    valueType r0, r1;
    indexType nrts;
    bool      cplx;
    bool      dblx;

    void findRoots();

  public:

    Quadratic() : nrts(0), cplx(false), dblx(false) {}

    Quadratic( valueType a, valueType b, valueType c )
    : nrts(0), cplx(false), dblx(false) {
      valueType & A = ABC[0];
      valueType & B = ABC[1];
      valueType & C = ABC[2];
      A = a; B = b; C = c;
      findRoots();
    }

    void
    setup( valueType a, valueType b, valueType c ) {
      valueType & A = ABC[0];
      valueType & B = ABC[1];
      valueType & C = ABC[2];
      A = a; B = b; C = c;
      findRoots();
    }

    indexType numRoots() const { return nrts; }

    bool complexRoots() const { return cplx; }

    bool doubleRoot() const { return dblx; }

    indexType getRealRoots( valueType r[] ) const;

    indexType getPositiveRoots( valueType r[] ) const;

    indexType getNegativeRoots( valueType r[] ) const;

    indexType getRootsInRange( valueType a, valueType b, valueType r[] ) const;

    indexType getRootsInOpenRange( valueType a, valueType b, valueType r[] ) const;

    valueType real_root0() const { return r0; }

    valueType real_root1() const { return r1; }

    complexType
    root0() const
    { return cplx ? complexType(r0,r1) : complexType(r0,0); }

    complexType
    root1() const
    { return cplx ? complexType(r0,-r1) : complexType(r1,0); }

    void
    getRoot0( valueType & re, valueType & im ) const {
      if ( cplx ) { re = r0; im = r1; }
      else        { re = r0; im = 0;  }
    }

    void
    getRoot0( complexType & r ) const
    { r = cplx ? complexType(r0,r1) : complexType(r0,0); }

    void
    getRoot1( valueType & re, valueType & im ) const {
      if ( cplx ) { re = r0; im = -r1; }
      else        { re = r1; im = 0;   }
    }

    void
    getRoot1( complexType & r ) const
    { r = cplx ? complexType(r0,-r1) : complexType(r1,0); }

    valueType
    eval( valueType x ) const
    { return evalPoly( ABC, 2, x ); }

    complexType
    eval( complexType const & x ) const
    { return evalPolyC( ABC, 2, x ); }

    void eval( valueType x, valueType & p, valueType & dp ) const;

    void
    info( std::ostream & s ) const;

    bool
    check( std::ostream & s ) const;

  };

  // - - - - - - - - - - - - - - - - - - - - - - - - - - - - - - - - - - -
  /*\
   |    ____      _     _
   |   / ___|   _| |__ (_) ___
   |  | |  | | | | '_ \| |/ __|
   |  | |__| |_| | |_) | | (__
   |   \____\__,_|_.__/|_|\___|
   |
   |  A * x^3 + B * x^2 + C * x + D
  \*/
  class Cubic {
    valueType ABCD[4];
    valueType r0, r1, r2;
    indexType nrts, iter;
    bool      cplx; // complex root
    bool      dblx; // double root
    bool      trpx; // triple root

    void findRoots();

  public:

    Cubic() : nrts(0), iter(0), cplx(false), trpx(false) {}
    Cubic( valueType _a, valueType _b, valueType _c, valueType _d )
    : nrts(0), iter(0), cplx(false), trpx(false) {
      valueType & A = ABCD[0];
      valueType & B = ABCD[1];
      valueType & C = ABCD[2];
      valueType & D = ABCD[3];
      A = _a; B = _b; C = _c; D = _d;
      findRoots();
    }

    void
    setup( valueType a, valueType b, valueType c, valueType d ) {
      valueType & A = ABCD[0];
      valueType & B = ABCD[1];
      valueType & C = ABCD[2];
      valueType & D = ABCD[3];
      A = a; B = b; C = c; D = d;
      findRoots();
    }

    indexType numRoots()     const { return nrts; }

    bool complexRoots() const { return cplx; }

    bool doubleRoot() const { return dblx; }

    bool tripleRoot() const { return trpx; }

    indexType getRealRoots( valueType r[] ) const;

    indexType getPositiveRoots( valueType r[] ) const;

    indexType getNegativeRoots( valueType r[] ) const;

    indexType getRootsInRange( valueType a, valueType b, valueType r[] ) const;

    indexType getRootsInOpenRange( valueType a, valueType b, valueType r[] ) const;

    valueType real_root0() const { return r0; }

    valueType real_root1() const { return r1; }

    valueType real_root2() const { return r2; }

    complexType
    root0() const
    { return cplx ? complexType(r0,r1) : complexType(r0,0); }

    complexType
    root1() const
    { return cplx ? complexType(r0,-r1) : complexType(r1,0); }

    complexType
    root2() const
    { return complexType(r2,0); }

    void
    getRoot0( valueType & re, valueType & im ) const {
      if ( cplx ) { re = r0; im = r1; }
      else        { re = r0; im = 0;  }
    }

    void
    getRoot0( complexType & r ) const
    { r = cplx ? complexType(r0,r1) : complexType(r0,0); }

    void
    getRoot1( valueType & re, valueType & im ) const {
      if ( cplx ) { re = r0; im = -r1; }
      else        { re = r1; im = 0;   }
    }

    void
    getRoot1( complexType & r ) const
    { r = cplx ? complexType(r0,-r1) : complexType(r1,0); }

    void
    getRoot2( valueType & re, valueType & im ) const
    { re = r2; im = 0; }

    void
    getRoot2( complexType & r ) const
    { r = complexType(r2,0); }

    valueType
    eval( valueType x ) const
    { return evalPoly( ABCD, 3, x ); }

    complexType
    eval( complexType const & x ) const
    { return evalPolyC( ABCD, 3, x ); }

    void eval( valueType x, valueType & p, valueType & dp ) const;

    void
    info( std::ostream & s ) const;

    bool
    check( std::ostream & s ) const;

  };

  // - - - - - - - - - - - - - - - - - - - - - - - - - - - - - - - - - - -
  /*\
   |    ___                   _   _
   |   / _ \ _   _  __ _ _ __| |_(_) ___
   |  | | | | | | |/ _` | '__| __| |/ __|
   |  | |_| | |_| | (_| | |  | |_| | (__
   |   \__\_\\__,_|\__,_|_|   \__|_|\___|
   |
   |  A * x^3 + B * x^2 + C * x + D
  \*/
  class Quartic {
    valueType ABCDE[5];
    valueType r0, r1, r2, r3;
    indexType iter, nreal, ncplx;

    void findRoots();

    bool cplx0() const { return ncplx > 0; }
    bool cplx1() const { return ncplx > 0; }
    bool cplx2() const { return ncplx > 2; }
    bool cplx3() const { return ncplx > 2; }

  public:

    Quartic() : iter(0), nreal(0), ncplx(0) {}
    Quartic(
      valueType _a,
      valueType _b,
      valueType _c,
      valueType _d,
      valueType _e
    )
    : iter(0), nreal(0), ncplx(0) {
      valueType & A = ABCDE[0];
      valueType & B = ABCDE[1];
      valueType & C = ABCDE[2];
      valueType & D = ABCDE[3];
      valueType & E = ABCDE[4];
      A = _a; B = _b; C = _c; D = _d; E = _e;
      findRoots();
    }

    void
    setup(
      valueType a,
      valueType b,
      valueType c,
      valueType d,
      valueType e
    ) {
      valueType & A = ABCDE[0];
      valueType & B = ABCDE[1];
      valueType & C = ABCDE[2];
      valueType & D = ABCDE[3];
      valueType & E = ABCDE[4];
      A = a; B = b; C = c; D = d; E = e;
      findRoots();
    }

    indexType numRoots() const { return nreal+ncplx; }

    indexType numRealRoots() const { return nreal; }

    indexType numComplexRoots() const { return ncplx; }

    indexType getRealRoots( valueType r[] ) const;

    indexType getPositiveRoots( valueType r[] ) const;

    indexType getNegativeRoots( valueType r[] ) const;

    indexType getRootsInRange( valueType a, valueType b, valueType r[] ) const;

    indexType getRootsInOpenRange( valueType a, valueType b, valueType r[] ) const;

    valueType real_root0() const { return r0; }

    valueType real_root1() const { return r1; }

    valueType real_root2() const { return r2; }

    valueType real_root3() const { return r3; }

    complexType
    root0() const
    { return cplx0() ? complexType(r0,r1) : complexType(r0,0); }

    complexType
    root1() const
    { return cplx1() ? complexType(r0,-r1) : complexType(r1,0); }

    complexType
    root2() const
    { return cplx2() ? complexType(r2,r3) : complexType(r2,0); }

    complexType
    root3() const
    { return cplx3() ? complexType(r2,-r3) : complexType(r3,0); }

    void
    getRoot0( valueType & re, valueType & im ) const {
      if ( cplx0() ) { re = r0; im = r1; }
      else           { re = r0; im = 0;  }
    }

    void
    getRoot0( complexType & r ) const {
      if ( cplx0() ) r = complexType(r0,r1);
      else           r = complexType(r0,0);
    }

    void
    getRoot1( valueType & re, valueType & im ) const {
      if ( cplx1() ) { re = r0; im = -r1; }
      else           { re = r1; im = 0;   }
    }

    void
    getRoot1( complexType & r ) const {
      if ( cplx1() ) r = complexType(r0,-r1);
      else           r = complexType(r1,0);
    }

    void
    getRoot2( valueType & re, valueType & im ) const {
      if ( cplx2() ) { re = r2; im = r3; }
      else           { re = r2; im = 0;  }
    }

    void
    getRoot2( complexType & r ) const {
      if ( cplx2() ) r = complexType(r2,r3);
      else           r = complexType(r2,0);
    }

    void
    getRoot3( valueType & re, valueType & im ) const {
      if ( cplx3() ) { re = r2; im = -r3; }
      else           { re = r3; im = 0;   }
    }

    void
    getRoot3( complexType & r ) const {
      if ( cplx3() ) r = complexType(r2,-r3);
      else           r = complexType(r3,0);
    }

    valueType
    eval( valueType x ) const
    { return evalPoly( ABCDE, 4, x ); }

    complexType
    eval( complexType const & x ) const
    { return evalPolyC( ABCDE, 4, x ); }

    void
    info( std::ostream & s ) const;

    bool
    check( std::ostream & s ) const;

  };

  /*\
   |   _   _ _   _ _
   |  | | | | |_(_) |___
   |  | | | | __| | / __|
   |  | |_| | |_| | \__ \
   |   \___/ \__|_|_|___/
  \*/

  #ifndef DOXYGEN_SHOULD_SKIP_THIS

  // x^3 + a*x^2 + b*x + c
  static
  inline
  valueType
  evalMonicCubic(
    valueType x,
    valueType a,
    valueType b,
    valueType c
  ) {
    valueType p;
    p = x + a;
    p = p * x + b;
    p = p * x + c;
    return p;
  }

  static
  inline
  void
  evalMonicCubic(
    valueType   x,
    valueType   a,
    valueType   b,
    valueType   c,
    valueType & p,
    valueType & dp
  ) {
    p  = x + a;
    dp = x + p;
    p  = p  * x + b;
    dp = dp * x + p;
    p  = p  * x + c;
  }

  // 3*x^2 + 2*a*x + b
  // 6*x + 2*a
  static
  inline
  void
  evalMonicCubic(
    valueType   x,
    valueType   a,
    valueType   b,
    valueType   c,
    valueType & p,
    valueType & dp,
    valueType & ddp
  ) {
    p   = x + a;
    dp  = x + p;      // 2*x + a
    p   = p  * x + b; // x^2 + a * x + b
    ddp = 2*(x + dp);
    dp  = dp * x + p;
    p   = p  * x + c;
  }

  // x^4 + a*x^3 + b*x^2 + c*x + d
  static
  inline
  valueType
  evalMonicQuartic(
    valueType x,
    valueType a,
    valueType b,
    valueType c,
    valueType d
  ) {
    valueType p;
    p = x + a;     // x + a
    p = p * x + b; // x^2+ a*x + b
    p = p * x + c; // x^3+ a*x^2 + b*x + c
    p = p * x + d; // x^4+ a*x^3 + b*x^2 + c*x + d
    return p;
  }

  static
  inline
  void
  evalMonicQuartic(
    valueType   x,
    valueType   a,
    valueType   b,
    valueType   c,
    valueType   d,
    valueType & p,
    valueType & dp
  ) {
    p  = x + a;      // x + a
    dp = x + p;      // 2*x + a
    p  = p  * x + b; // x^2+ a*x + b
    dp = dp * x + p; // 3*x^2 + 2*a*x + b
    p  = p  * x + c; // x^3+ a*x^2 + b*x + c
    dp = dp * x + p; // 4*x^3 + 3*a*x^2 + 2*b*x + c
    p  = p  * x + d; // x^4+ a*x^3 + b*x^2 + c*x + d
  }

  static
  inline
  void
  evalMonicQuartic(
    valueType   x,
    valueType   a,
    valueType   b,
    valueType   c,
    valueType   d,
    valueType & p,
    valueType & dp,
    valueType & ddp
  ) {
    // p_{n+1}(x)   = x * p_{n}(x) + b_{n}
    // p'_{n+1}(x)  = x * p'_{n}(x) + p_{n}(x)
    // p''_{n+1}(x) = x * p''_{n}(x) + 2*p'_{n}(x)
    // ddp = 0;
    // dp  = 1;
    p   = x + a;     // x + a

    ddp = 2;
    dp  = x + p;
    p   = p * x + b;

    ddp = ddp * x + 2 * dp;
    dp  = dp * x + p;
    p   = p * x + c;

    ddp = ddp * x + 2 * dp;
    dp  = dp * x + p;
    p   = p * x + d;
  }

  #endif

}

#endif
```

### Quick search

### Table of Contents

- Quartic Roots Solver
- C++ API

«
hide menu

menu
sidebar
»

### Navigation

- index
- toc
- Quartic Roots »
- Program Listing for File PolynomialRoots.hh

© Copyright 2021, Enrico Bertolazzi.
Created using Sphinx 4.2.0.
